# Assessing the climate suitability and potential economic impacts of Oak wilt in Canada

**DOI:** 10.1038/s41598-020-75549-w

**Published:** 2020-11-10

**Authors:** John H. Pedlar, Daniel W. McKenney, Emily Hope, Sharon Reed, Jon Sweeney

**Affiliations:** 1 Canadian Forest Service, Great Lakes Forestry Centre, Natural Resources Canada, 1219 Queen Street East, Sault Ste. Marie, ON Canada; 2 Ontario Forest Research Institute, 1235 Queen Street East, Sault Ste. Marie, ON Canada; 3 Canadian Forest Service, Atlantic Forestry Centre, Natural Resources Canada, 1350 Regent Street, Fredericton, New Brunswick Canada

**Keywords:** Environmental health, Invasive species, Environmental economics

## Abstract

We assess risks posed by oak wilt—a disease caused by the fungal pathogen *Bretziella fagacearum*. Though not currently found in Canada, our distribution models indicate that suitable climate conditions currently occur in southern Ontario for *B. fagacearum* and two of its main insect dispersal vectors, *Colopterus truncatus* and *Carpophilus sayi.* Climate habitat for these species is projected to expand northward under climate change, with much of the oak range in eastern Canada becoming climatically suitable within the next two decades. Potential costs for the removal and replacement of oak street trees ranged from CDN$266 to $420 million, with variation related to uncertainty in costs, rate of tree replacement, and city-level estimates of oak street tree density. The value of standing oak timber in eastern Canada was estimated at CDN$126 million using provincial stumpage fees and as a CDN$24 million annual contribution to national Gross Domestic Product (GDP) when calculated using a combination of economic and forestry product statistics. These values can help inform the scale of eradication and/or management efforts in the event of future oak wilt introductions.

## Introduction

Oak wilt, caused by the fungal pathogen *Bretziella fagacearum* (formerly *Ceratocystis fagacearum*)^1^, is a significant disease of oaks (*Quercus* spp.) in the United States^2^, and a growing concern to Canadian regulators of plant and forest health^3^. The disease manifests when *B. fagacearum* becomes established in the sapwood of a host tree, causing the tree to produce protective tissues and gums that restrict the flow of water and nutrients^4^. The tree subsequently wilts and dies. For members of the red oak group (Section *Lobatae*), this process can occur within weeks, while for members of the white oak group (Section *Quercus*) disease progression and ultimate outcome are less predictable^2^.

*Bretziella fagacearum* was first documented in Wisconsin^5^ and is currently present in 24 midwestern and eastern states^6^. However, historical accounts suggest that the pathogen may have been present in the United States as early as the late 1800s^5^. Geographic origins of *B. fagacearum* are uncertain, but genetic data suggest that it was brought to the United States in a single introduction event—possibly from Mexico or Central/Southern America^5^. To date, the pathogen has not been documented in Canada, but it is present in all states bordering the province of Ontario and has been reported from Belle Isle, Michigan—an island in the Detroit River that is within 600 m of the city of Windsor, Ontario^7^.

Oak wilt can be transmitted by a number of mechanisms. Below-ground transmission occurs when oak trees in close proximity to one another form root grafts that allow the movement of xylem contents between trees^2^. In pure oak stands, such connections can result in an expansion of the disease in a series of concentric rings. The root systems of infected trees survive for several years and are able to graft to healthy trees that are expanding their root systems^8^. This results in new outbreaks as saplings, stump, and root sprouts succumb to the disease. Above-ground transmission, also called overland spread, occurs primarily via insect vectors, which acquire *B. fagacearum* spores while feeding and/or ovipositing at the site of fungal mats formed under the bark by the expanding pathogen. Nitidulid beetles (Family Nitidulidae), such as *Colopterus truncatus* and *Carpophilus sayi*, appear to be the main insect vectors, while oak bark beetles (e.g., *Pseudopityophthorus* spp.) appear to play a minor role^2^. Since nitidulid beetles do not bore into trees, they require fresh xylem-penetrating wounds in recipient trees in order to infect a new host. Under natural conditions, these vectors are thought to be able to move *B. fagacearum* up to several kilometres in a year^5^. However, human transport of contaminated logs can result in long distance movement of the pathogen; such movements have been implicated in the appearance of *B. fagacearum* on the Upper Peninsula of Michigan in the 1970s and in its spread across Texas^5^.

Concern regarding the destructive nature of oak wilt has resulted in numerous programs to prevent new infections and manage expanding epicenters. Prevention of overland spread is considered highly effective^9^. This strategy requires identifying a high-risk time period, typically April–July in the Midwestern US, when nitidulid beetles are actively flying and infecting trees^10^. The public is encouraged to avoid tree wounding during this high-risk period or apply wound dressings to prevent infections. Once oak wilt is established at a location, tools available for management include root disruption, sanitation, and chemical applications^9^. The tools used depend on the management goals. Most programs first disrupt root grafts to stop spread using heavy equipment such as a vibratory plow. Water permeable barriers can be placed in trenches to stop re-grafting, thereby reducing the need to return and repeat root disruption methods^11^. Sanitation (e.g. cutting, chipping, covering logs) typically follows root disruption, with the goal of preventing infected trees from producing fungal mats or insects from accessing fungal mats. Asymptomatic trees next to oak wilt killed trees are also removed because they are usually infected belowground and develop symptoms the following year. Chemical therapeutic treatments can be used to prevent or treat symptoms, but differ in their success rates for white and red oaks and must be repeated on a regular basis^9^. Girdling and herbicide treatments, paired with early detection, are used in some management programs, but herbicides do not immediately kill all roots or stop belowground transmission^8^.

Oaks (Genus: *Quercus*) are an important group of trees in Canada, where they are represented by nine species in the eastern portion of the country (several of which are found only in southern Ontario) and one species (*Quercus garryana*) in southern British Columbia^12^. Summaries derived from Canada’s National Forest Inventory^13^ indicate that total oak volume in Canada is approximately 14.2 million m^3^, with peak abundance in southcentral Ontario and southern Québec. In natural forest settings, oaks are often found in mixture with other broadleaved species and occupy a range of sites including swamps (e.g., *Q. bicolor*), rich bottomlands (e.g., *Q. macrocarpa*), and dry ridges (e.g. *Q. rubra*). Oak wood, known for its strength and durability, is used primarily in furniture and flooring, while acorns act as a food source for a variety of wildlife species. Oaks are also an important street and landscape tree in many Canadian urban areas^14^.

Understanding the climatic suitability of an area for a potentially invasive species is important because it allows for an assessment of values that may be placed at risk if the species becomes established there. Here we assess the climatic suitability of Canada, under both current and future climate, for *B. fagacearum* and two of its main insect vectors—*C. truncatus* and *C. sayi*. We then assess several potential economic impacts of the disease, including costs related to the removal and replacement of high-value street trees and lost forestry revenues. As with other invasive, non-native species, detailed depictions of likely economic outcomes are problematic given the various uncertainties of establishment, spread, and impact through time^15^. Nevertheless, broad-scale perspectives such as these can provide important contextual information to support investments for prevention and preparedness, including research initiatives^16^.

## Methods

### Species distribution modelling

**Figure 1:**
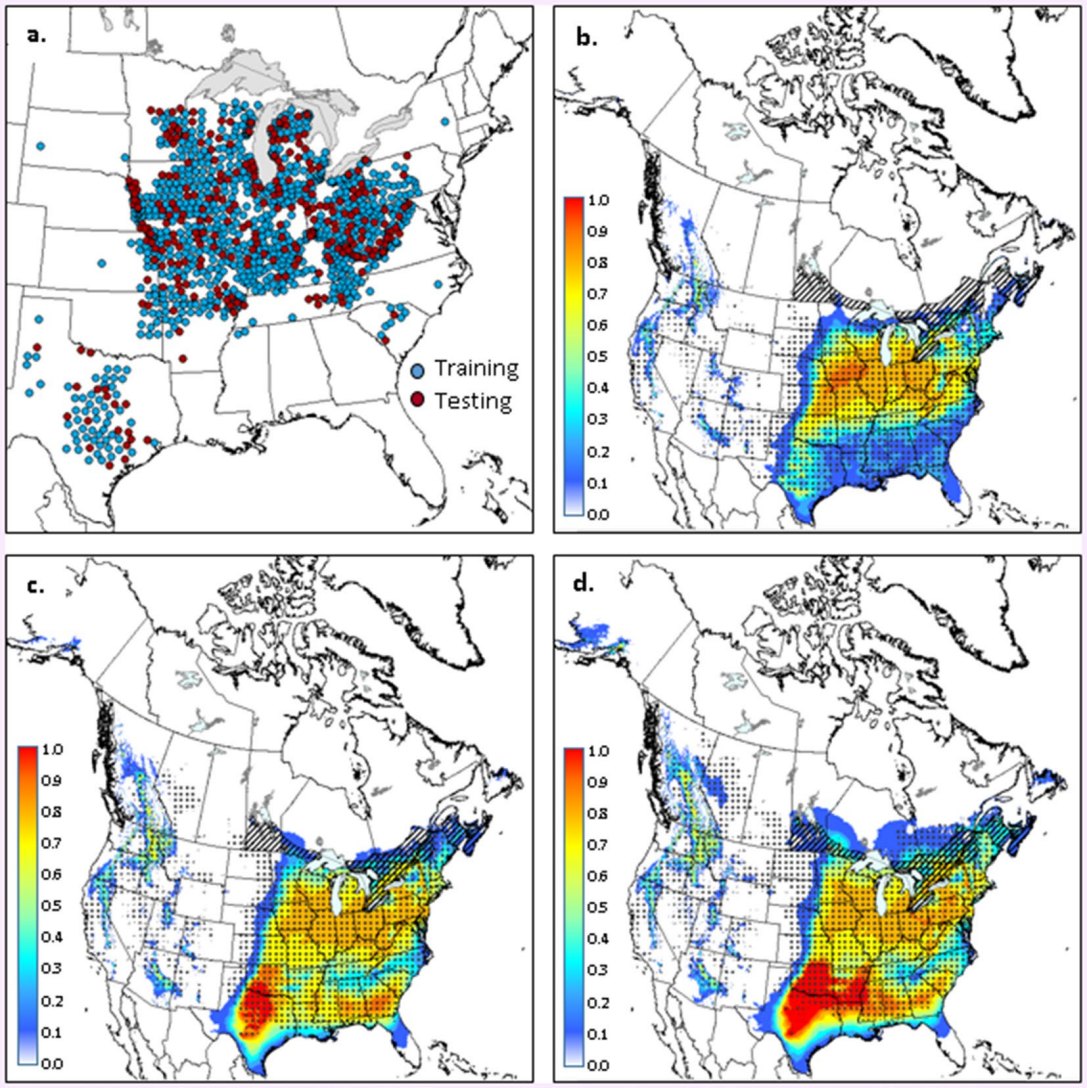
Occurrence data (**a**) used for generating climate suitability models for *Bretziella fagacearum*. Maps with colour gradients indicate Maxent-derived climate suitability for *B. fagacearum* for the: 1981–2010 period (**b**); 2011–2040 period (**c**); and 2041–2070 period (**d**). Stippling delineates the ANUCLIM-derived climate envelope for *B. fagacearum* in each time period. Hatching delineates the current distribution of *Quercus* in Canada. Climate projections are based on a composite of four climate models and the RCP 4.5 emissions scenario (see text for further details). Maps were generated using ARCGIS v.9.3 (ESRI, Redlands, CA, USA; https://www.esri.com/arcgis/about-arcgis).

**Figure 2:**
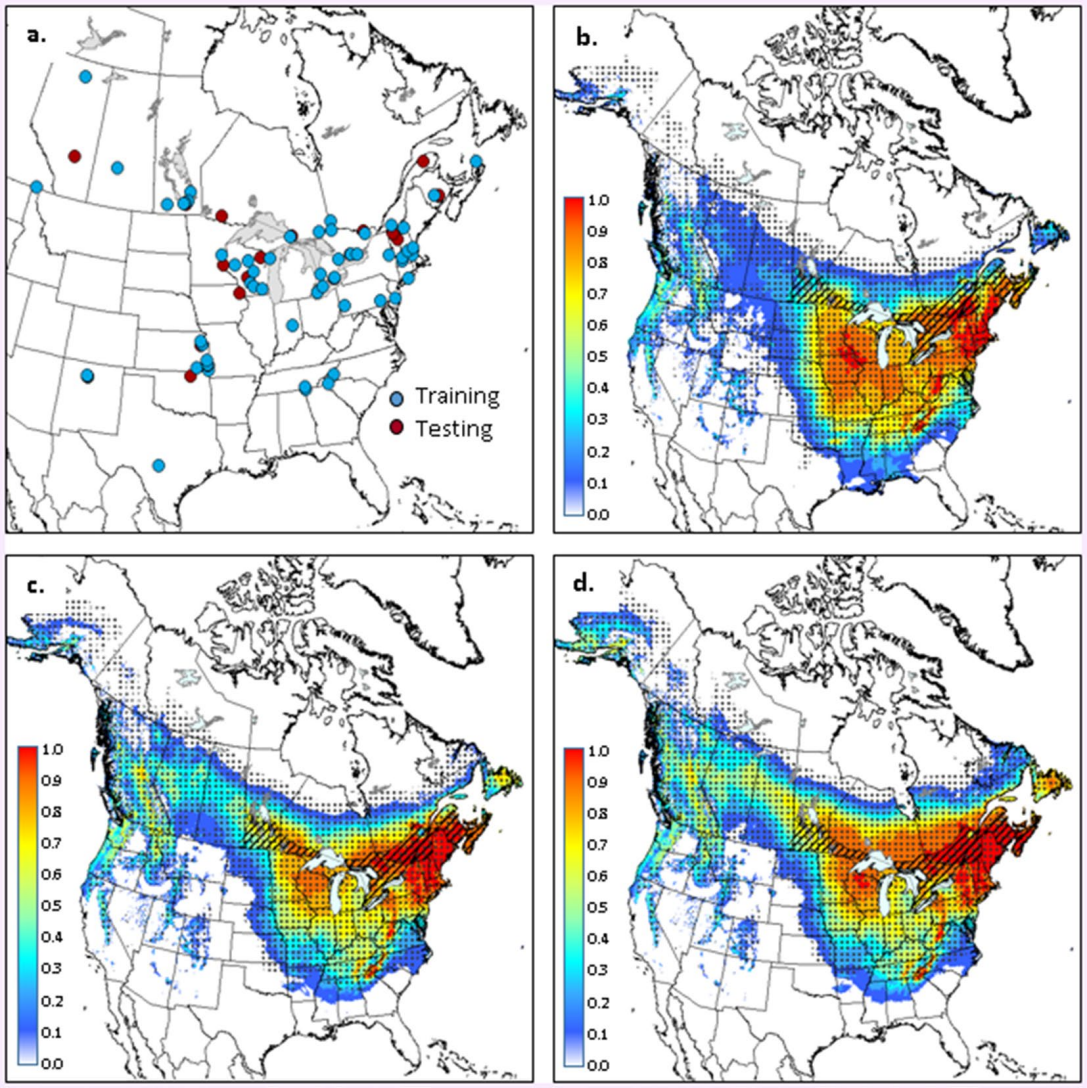
Occurrence data (**a**) used for generating climate suitability models for *Colopterus truncatus*. Maps with colour gradients indicate Maxent-derived climate suitability for C*. truncatus* for the: 1981–2010 period (**b**); 2011–2040 period (**c**); and 2041–2070 period (**d**). Stippling delineates the ANUCLIM-derived climate envelope for *C. truncatus* in each time period. Hatching delineates the current distribution of *Quercus* in Canada. Climate projections are based on a composite of four climate models and the RCP 4.5 emissions scenario (see text for further details). Maps were generated using ARCGIS v.9.3 (ESRI, Redlands, CA, USA; https://www.esri.com/arcgis/about-arcgis).

**Figure 3:**
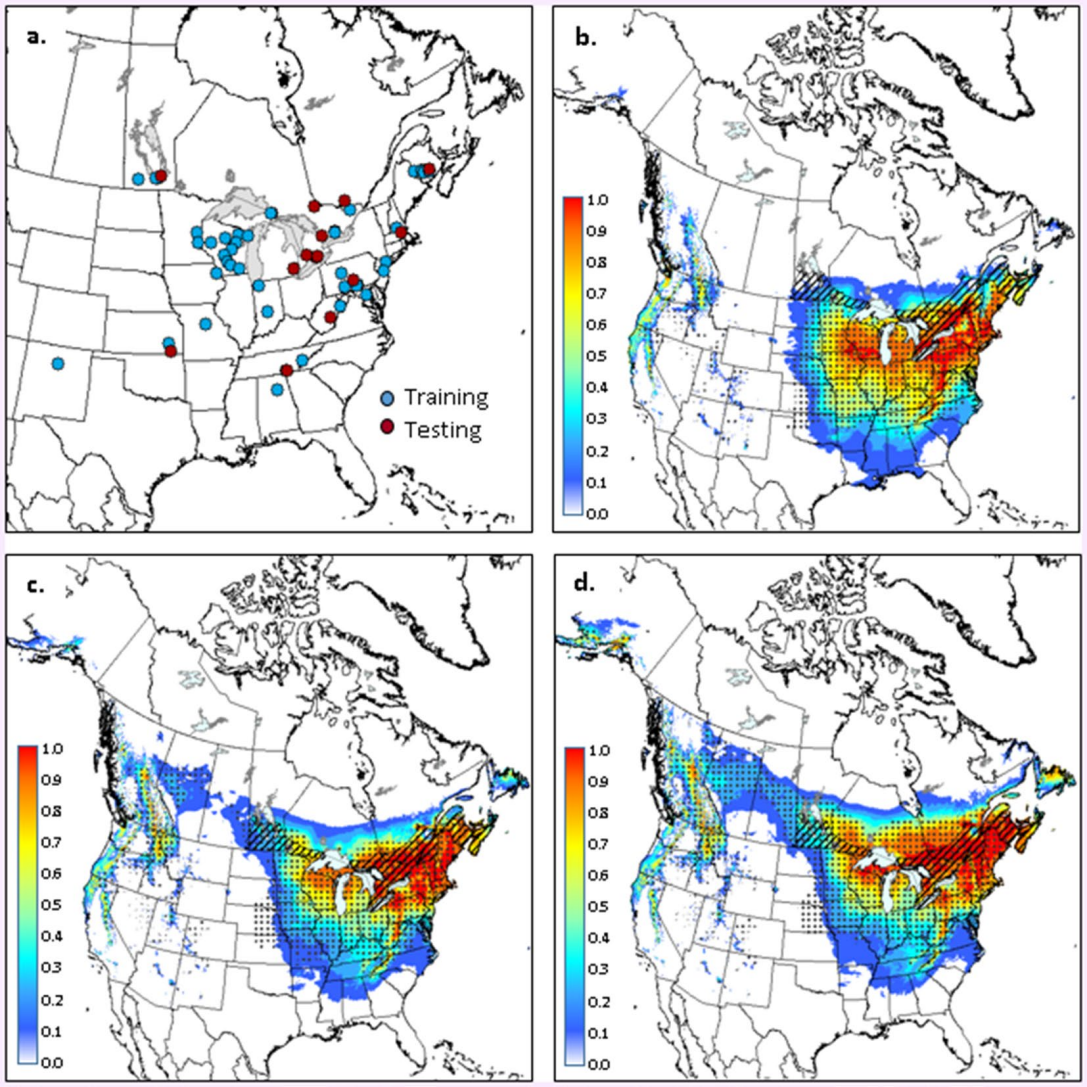
Occurrence data (**a**) used for generating climate suitability models for *Carpophilus sayi*. Maps with colour gradients indicate Maxent-derived climate suitability for C*. sayi* for the: 1981–2010 period (**b**); 2011–2040 period (**c**); and 2041–2070 period (**d**). Stippling delineates the ANUCLIM-derived climate envelope for *C. sayi* in each time period. Hatching delineates the current distribution of *Quercus* in Canada. Climate projections are based on a composite of four climate models and the RCP 4.5 emissions scenario (see text for further details). Maps were generated using ARCGIS v.9.3 (ESRI, Redlands, CA, USA; https://www.esri.com/arcgis/about-arcgis).

A total of 1548 occurrence locations were obtained for *B. fagacearum* from the United States Forest Service (provided to us by Erin Bullas-Appleton of the Canadian Food Inspection Agency in July 2018) and the Global Biodiversity Information Facility^17^. These records were filtered at a 300 arcsecond (approximately 10-km) resolution to remove duplicates and reduce spatial clustering, leaving 1401 unique location records (Fig. 1a). Occurrence locations for two key insect vectors of oak wilt, *C. truncatus* (Fig. 2a) and *C. sayi* (Fig. 3a)*,* were obtained from GBIF, publications^10,18^, and from specimens in the following collections: Atlantic Forestry Centre, Fredericton, NB, Canada; Canadian National Collection of Insects, Arachnids, and Nematodes, Agriculture and Agri-Food Canada Research Centre, Ottawa, ON, Canada; Ontario Forest Research Institute, Sault Ste. Marie, ON, Canada; Gareth S. Powell Collection, Nephi, UT, USA; Reginald Webster Collection, Charters Settlement, New Brunswick, Canada; and the Florida State Collection of Arthropods, Gainesville, FL, USA . After filtering at a 10-km resolution, there were 82 and 58 unique occurrence records for *C. truncatus* and *C. sayi* respectively.

Climate estimates were obtained at each occurrence location by interrogating North American climate models (described in McKenney et al.^19^) of the 1981–2010 normal period for the following four variables: (1) fall (i.e., September–December) precipitation (FALLPCP); (2) average spring (i.e., March–June) temperature (SPRINGTMP); (3) annual climate moisture index (CMI; a climate-based moisture balance variable; see Hogg^20^ for details); and (4) Average Minimum Temperature of the Coldest Month (MINTCM). These climate variables were selected based on their reported influences on *B. fagacearum* (FALLPCP and CMI^21,22^) *C. sayi* and *C. truncatus* (SPRINGTMP^10^), and insect distributions in general (MINTCM^23^). Further, none of the selected variables were highly correlated (i.e., r < 0.7 in all pairwise comparisons), thus alleviating concerns regarding the impact of collinearity among environmental variables on species distribution models^24^.

Future climate habitat maps for the 2011–2040 and 2041–2070 periods were generated using projections of the four climate variables described above from a composite (i.e., average) of four Earth System Models (ESMs): CanESM2, CESM1CAM5, HadGEM2-ES, and MIROC-ESM (see Price et al.^25^ for details on these models and the downscaling approach used). All projections employed a moderate greenhouse gas emissions scenario (i.e., RCP4.5; van Vuuren et al.^26^), which incorporates expected reductions in future greenhouse gas emissions and best describes the path of recent emissions^27^.

Spatial predictions of the potential distribution of *B. fagacearum* and its two main insect vectors in North America were generated using Maxent^28^—a machine learning method that estimates the distribution of a species by finding the distribution of maximum entropy subject to a set of spatial constraints defined by the environmental conditions at the occurrence locations. Maxent employs a regularization parameter (set to the default value of 1 for the current work), which determines the smoothness of the resulting models, and a variety of response functions (i.e., linear, product, quadratic, hinge, threshold, and categorical) to model potentially complex occurrence-environment relationships.

The selection of background points is an important component of a Maxent analysis as this provides a null distribution against which the occurrence locations are compared. Phillips^29^ recommended that background data points be selected from the same general area as the occurrence observations. However, in our experience, selecting background points that are too close to occurrence locations can also produce flawed results as the machine learning algorithm struggles to distinguish between suitable and unsuitable environmental conditions. Given that we are modelling three species whose geographic limits are unknown (and for which occurrence data is likely incomplete), we felt it was appropriate to consider a somewhat wider environmental domain. Thus, we selected 10,000 random background points from within the treed ecosystems of North America by masking out the Arctic Cordillera, Tundra, and North American Desert ecoregions (based on Level 1 ecoregion definitions by the Commission for Environmental Cooperation^30^) from the domain used for background point selection.

Assessing the performance of Maxent models has been a somewhat contentious issue. The area under the receiver operator curve (AUC) statistic, which is the default assessment metric provided with the Maxent software, purports to provide a threshold-independent measure of predictive accuracy based on the ranking of locations^31^. This metric, which ranges from 0 to 1, can be interpreted as the probability that a randomly chosen presence location is ranked higher than a randomly chosen background point. However, AUC has been criticized for producing overly optimistic measures of fit when background points are taken from extensive, ecologically unviable locations^32^. Alternative metrics, such as the True Skill Statistic (TSS^33^) require the definition of a suitable threshold to convert Maxent output to a binary (presence/absence) spatial product, and then employ background points to calculate the standard components of a confusion matrix (e.g., true positives, false positives, etc.). This approach has also been criticized, as confusion matrices are more appropriately constructed using true absence data—particularly for species with low sampling effort and/or those lacking a stable geographic distribution^31^. Recently, Wunderlich et al.^34^ proposed the repurposing of a metric commonly used in meteorology called the Symmetric Extremal Dependence Index (SEDI) to assess the accuracy of species distribution models. This metric, which makes use of logged confusion matrix components and ranges between − 1 and 1, was shown to outperform TSS under a range of modelling outcomes, particularly when background points greatly outnumber occurrence locations as is the case in the current study^34^. Thus, we present two performance metrics for the current work: AUC (the most commonly reported method for assessing Maxent performance) and SEDI (a promising new performance metric). These metrics were calculated using a random sample of 25% of the occurrence data that was withheld from the model training process. Note that, in order to produce the strongest final distribution maps, final models were run with the full set of occurrence locations; thus the performance metrics based on withheld data should be considered minimum estimates of the predictive accuracy of the model. In order to convert Maxent predictions into binary outcomes for calculating SEDI, we employed the ‘balanced’ threshold, which is provided as a standard output with each Maxent model. Specifically, grid cells with climate suitability less than 0.05, 0.05, and 0.06 were defined as unsuitable for *B. fagacearum*, *C. truncatus*, and *C. sayi* respectively.

As a complimentary modelling approach, climate envelopes were generated for each organism and time period of interest using the ANUCLIM software system^35,36^. This system, which represents an early generation, but still useful, tool for species distribution modelling, provides statistical summaries (i.e., min, max, mean, and various percentiles) for each climate variable of interest based on the distribution of values across occurrence locations. Climate envelopes can be defined for the full range of climate conditions at which a species is known to occur (i.e., using min/max values) or for a core range of conditions (e.g., 5th and 95th percentiles). An overall climate envelope is then defined by intersecting the envelopes for each climate variable of interest. For the current work, climate envelopes were defined based on the minimum and maximum values obtained at occurrence locations for each of the four climate variables described above. Final envelopes were overlaid on the Maxent-based climate suitability maps to provide a further assessment of the reliability of these outputs.

To assist in assessing risk to Canadian forests, the geographic range of oak in Canada was delineated by carrying out a geometric union (in ArcGIS v 10.4.1) of the individual ranges of the ten oak species that occur in Canada. For this analysis, we employed digital versions of Little’s (1971) North American tree range maps^37^. Projections of oak migration under climate change were not incorporated here as we anticipate minimal tree migration by the middle of the current century^38^.

### Potential impacts on street trees

Street tree information was obtained from a survey that has been described previously^39^. The original survey involves participants walking (or driving) a number of 0.5-km long spatially randomized routes in an urban area. During a survey, each tree within 10 m of the road is identified to genus (or species if possible) and classified according to height (i.e., small ≤ 5 m; medium = 5–10 m; large ≥ 10 m). The number of survey routes in each community was determined such that tree densities could be estimated within reasonable error bounds: ± 5 trees/km for common species and ± 1 tree/km for uncommon species; typically, routes covered 5–10% of total road length in each community^39^. Canadian Forest Service staff and volunteers employed this approach to collect information on street tree composition for 53 communities in eastern Canada between 2009 and 2015.

A variation on this approach, in which surveys were carried out using Google StreetView imagery, was introduced in 2016. Surveys in 49 communities have since been completed using this approach. Follow-up ground surveys indicated that the StreetView-based surveys were 89% accurate at the genus level and 66% at the species level (unpublished data). These numbers are comparable to other Google-based tree surveys^40^. Note that, for the current work, the genus-level accuracy is most relevant given that *B. fagacearum* attacks all oak species.

Based on the surveys described above, we collected tree composition and size data for 106 urban centers in eastern Canada. For each of these centers, we calculated oak frequency per kilometer of urban roadway in each size class. Street tree density values were assigned to the remaining urban centers in the study area using an inverse-distance weighted average of the five nearest communities with survey data. Finally, we estimated the total number of host trees in each size class in each community by multiplying the community-level oak density values by the length of urban roadway in each community.

Tree removal and replacement costs (and ranges) were obtained through consultation with tree care professionals and municipal foresters (Table 1). We developed a spreadsheet model to calculate costs for each urban center and for the study area as a whole. Uncertainty in parameter values was explored using @Risk, a spreadsheet add-on that enables detailed Monte Carlo simulations^41^. Specifically, variation in tree removal and replacement costs was explored using a triangular distribution with parameters shown in Table 1, while variation in tree replacement was explored using rates of 0%, 50%, and 100%.

**Table 1:** Removal and replacement cost estimates for each tree size class (CDN$).

Cost category	Small (< 5 m high) ($)		Medium (5–10 m high) ($)		Large (> 10 m high) ($)	
	Mean	Range	Mean	Range	Mean	Range
Removal	300	150–500	500	200–1000	1500	900–2000
Replacement	400	360–440	400	360–440	400	360–440
Total	700	510–940	900	560–1440	1900	1260–2440

### Timber-related losses

One approach for placing an economic value on potential forest losses is to employ standing timber (or stumpage) values, which are the fees paid by forest companies (typically to a provincial government in Canada) for the rights to harvest trees on a given land base. To carry out such a valuation, we used national forest attribute maps^13^ to derive gross merchantable oak volumes for four age classes (0–20, 20–40, 40–60, and > 60 years) for each province in our study area. Focusing on current/near-term oak timber stocks (since we are not estimating *B. fagacearum* spread), we multiplied merchantable volume over 40 years old—roughly the age at which oak becomes harvestable in Ontario^42^—by average provincial stumpage values.

Estimating stumpage values was somewhat challenging due to inter-provincial variation in stumpage systems and reporting of stumpage fees. For the province of Québec, we obtained oak-specific stumpage fees for 191 harvest zones for the period April 1, 2019 to March 31, 2020 (Bureau de mise en marché^43^). Since stumpage fees in Québec vary by wood quality class (i.e., A, B, and C), we further obtained information on the proportion of wood harvested in each class over the same period (unpublished dataset, Bureau de mise en marché des Bois). We then calculated the average stumpage fee for the province by averaging across harvest zones and quality classes, while weighting by the proportion of wood in each quality class. For Ontario, we obtained stumpage fees for two quality classes of hardwoods (i.e., Class 1 and Class 2) and four oak-related product types (Veneer, Sawlogs, Composite, and Firewood) for January 1 to December 31, 2019 (Ontario Ministry of Natural Resources and Forestry^44^). Given that oak is typically considered a higher value hardwood, and in lieu of information on how oak is partitioned across product types in Ontario, we calculated oak stumpage fees as an average across product types for the Class 1 hardwood category. Note that the stumpage rates employed here include the Renewal and Forest Futures fees that are part of the Ontario stumpage system. Finally, stumpage values for the province of Nova Scotia were obtained for a single hardwood quality class for the period April 1, 2017 to March 31, 2018 (Province of Nova Scotia^45^). As in Ontario, stumpage fees were averaged across product types. The stumpage values for Nova Scotia were applied to the relatively small amount of oak in the neighbouring Maritime Provinces of New Brunswick and Prince Edward Island (PEI).

Alternatively, gross domestic product (GDP) can provide an estimate of the total economic activity associated with a given industry. Annual GDP estimates for broad categories (e.g., forestry and logging industry, and wood product manufacturing industry) are available for each province at Natural Resources Canada’s forestry statistics website (https://cfs.nrcan.gc.ca/statsprofile/overview/ca). In order to estimate GDP specifically for oak-related timber products, we first multiplied these provincial broad category GDP values by the proportion of the total provincial harvest that was composed of hardwoods (multipliers obtained from published provincial data sources as detailed in the Results section below). This value was then further refined by multiplying by the proportion of hardwoods in the province that was composed of oak species. These estimates were obtained from forest attribute grids^13^, by summing merchantable volume of (1) oak and (2) all broadleaf species within the industrial forestry limits of each province. Spatial summaries were carried out using the raster and rgdal packages in r. Though admittedly coarse, we felt this approach was the best available given the dearth of readily available economic data for individual tree species/genera; similar approaches have been used previously to estimate economic impacts of invasive species^46,47^.

These two approaches (i.e., stumpage-based and GDP-based) provide different perspectives on oak-related timber values at risk. The stumpage approach attaches a basic price to standing timber resources, but does not consider downstream economic activities associated with harvest, such as wages, equipment purchases, and capital expenditures. This approach implicitly assumes that substitution possibilities (e.g., other tree species) can fully replace oak-related contributions to the economy with minimal adjustment costs and, as such, is a conservative estimate of potential timber value losses. Alternatively, the GDP approach attempts to include all downstream economic contributions and assumes little or no opportunity for substitution, such that oak timber losses would be accompanied by a proportional reduction in economic activity. We present both estimates here to provide policy-makers with a range of possible impacts. The value of costs through time is generally arrived at using economic discounting; however, here we have no estimates of the timeline associated with oak wilt spread and hence have chosen to report gross, undiscounted values. See Aukema et al.^48^ for further discussion.

## Results

A preliminary Maxent run, with 25% of the *B. fagacearum* occurrence data withheld from the model building process, indicated high predictive capacity associated with the model (AUC = 0.93, SEDI = 0.95). Among the four climate variables, FALLPCP and SPRINGTMP were the most important, accounting for 41.2 and 35.6% of explained variance respectively. Each of the remaining climate variables accounted for less than 20% of explained variance, though correlations among variables make it challenging to definitively ascribe causation.

The potential distribution map indicated moderate to low climate suitability (index values of 0.1–0.5) for *B. fagacearum* across southern Ontario, southern Québec, and portions of the Maritime provinces over the 1981–2010 period (Fig. 1b). Areas of highest suitability (index values of 0.7–0.8) were located in far southern Ontario, near known *B. fagacearum* occurrence locations in southeastern Michigan. In the United States, climate suitability was high across many states in the northeastern portion of the country, with moderate suitability extending into Texas as well.

Agreement between the Maxent- and ANUCLIM-based predictions was high, with 69% overlap between the two approaches. The main area of disagreement was in the American Midwest, where the ANUCLIM prediction extended further west than that of Maxent (Fig. 1b). Importantly, overlap was nearly complete across southern Canada, lending further support to the notion that suitable climate habitat for *B. fagacearum* already exists in Canada.

Climate suitability was projected to increase in eastern Canada over the 2011–2040 and 2041–2070 periods (Fig. 1c,d). By the middle of the current century, both the Maxent- and ANUCLIM-based approaches projected suitable climate for *B. fagacearum* across much of the current range of oak in Canada. Overlap between the two approaches was 68% and 61% for the 2011–2040 and 2041–2070 periods respectively, with some lack of agreement in northern Ontario and Alberta, the American Midwest, and Texas.

Models for *C. truncatus* and *C. sayi* also showed good predictive capacity, with withheld AUC values of 0.89 and 0.93, respectively and SEDI values of 0.81 and 0.90, respectively. SPRINGTMP and FALLPCP were identified as key climate variables for both species, explaining 51 and 22% of variation respectively for *C. truncatus* and 43 and 28% of variation respectively for *C. sayi*. Climate suitability was high (values > 0.7) in southern Ontario, Québec and much of the Atlantic region during the 1981–2010 period (Figs. 2b, 3b). Similar to *B. fagacearum*, suitable climate was projected to move northward and westward, covering much of the range of oak in Canada by the middle of the current century (Figs. 2c,d; 3c,d). Overlap between Maxent and ANUCLIM projections ranged from 58 to 80% across species and time periods, with strong agreement across the central portion of projected ranges and some disagreement along southern and northern range limits.

Based on our urban tree surveys, there were 2.06 ± 1.36 (mean ± S.D.) oak trees per kilometer of urban roadway across our study area, with densities of 0.45 ± 0.33, 0.64 ± 0.42, and 0.97 ± 0.85 for small, medium, and large size classes respectively. We estimated 7.9 × 10^4^ small trees, 1.2 × 10^5^ medium-sized trees, and 1.6 × 10^5^ large trees, for a total of 3.5 × 10^5^ oak trees along city streets across the 485 urban areas in our study area.

**Figure 4:**
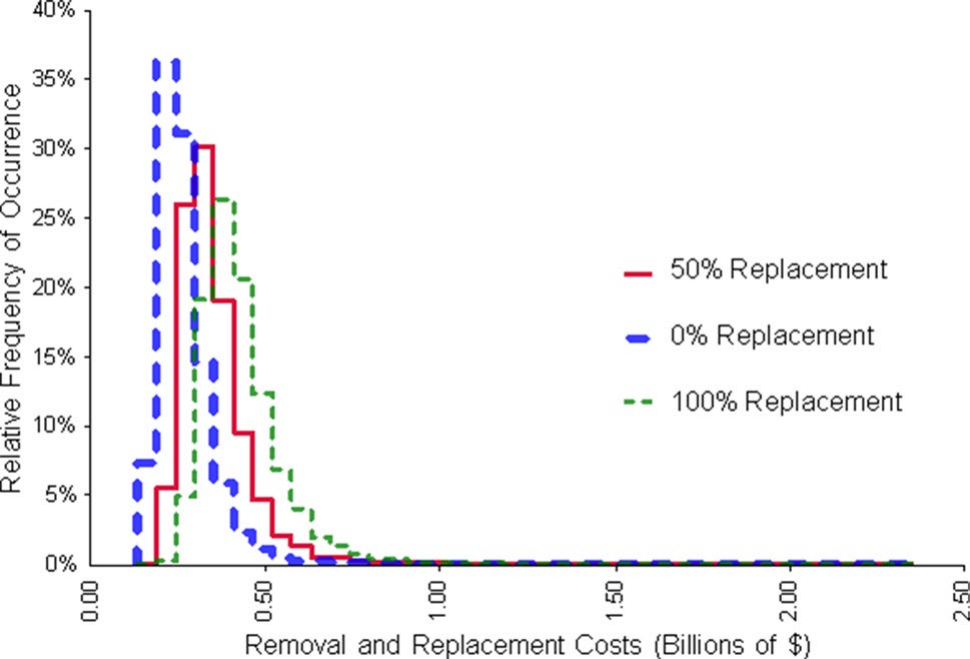
Potential costs (CDN$) associated with the removal and replanting of oak wilt host trees located near urban roadways in eastern Canada. The range in potential costs is derived from a Monte Carlo simulation that incorporated uncertainty in city-level oak street tree density estimates, removal and replanting costs, and three replanting scenarios wherein 0, 50, and 100% of trees were replaced.

Cost estimates related to the removal and replacement of oak trees along city streets are shown in Fig. 4. Based on replacement rates of 0, 50, and 100%, mean estimated costs across our study area were approximately CDN$266 million, CDN$349 million, and CDN$420 million respectively. City-level street tree costs varied in relation to city size and tree composition (Table 2 and Supp. Table S1). Based on default model inputs, Montreal, Québec had the highest potential impact value of any city in eastern Canada (CDN$53.7 million), followed by Toronto, Ontario (CDN$48.4 million), Halifax, Nova Scotia (CDN$13.0 million), Québec City, Québec (CDN$12.5 million), and Hamilton, Ontario (CDN$12.3 million). The average cost for all urban areas in our study was CDN$0.7 million, with the majority of values falling between CDN$0.05 million and CDN$1 million (Supp. Table S1).

**Table 2:** Top 20 urban areas in eastern Canada with respect to potential impacts (CDN$) of oak wilt on street trees.

Urban area	Province	Road length (km)	Host density (trees/km)			Potential impact ($ × 10^6^)
			Small	Med	Large	
Montréal	PQ	26,218	0.35	0.67	0.96	53.7
Toronto	ON	27,457	0.52	0.77	0.66	48.8
Halifax	NS	2929	0.36	1.21	2.34	13.0
Québec	PQ	7663	0.75	0.63	0.57	12.5
Hamilton	ON	5848	0.69	0.96	0.73	12.3
Winnipeg	MB	6032	0.02	0.23	1.09	10.5
Saint John	NB	1854	1.21	1.16	2.6	9.5
Kitchener	ON	3818	0.86	1.22	0.77	9.3
St. Catharines—Niagara	ON	4503	0.56	0.8	0.63	8.0
Ottawa—Gatineau	ON	5435	0.05	0.32	0.71	7.0
Oshawa	ON	2245	0.53	0.55	1.38	5.9
Barrie	ON	1868	0.58	0.6	1.64	5.7
London	ON	2976	0.68	0.98	0.5	5.3
Thunder Bay	ON	1498	0.04	0.35	1.61	3.9
Sherbrooke	PQ	1830	0.75	0.76	0.8	3.8
Windsor	ON	3022	0.42	0.59	0.28	3.2
Saint-Jean-sur-Richelieu	PQ	1201	0.4	0.75	1.09	2.8
North Bay	ON	821	0.4	0.52	1.84	2.6
Kentville	NS	277	1.16	2.27	4.85	2.5
Sault Ste. Marie	ON	795	0.07	0.49	1.95	2.5

When calculated using gross merchantable volume and provincial stumpage fees, oak timber values across the study area totaled CDN$126 million, with the highest values in Ontario, followed by Québec and the Maritimes region (Table 3). Alternatively, we used provincial gross domestic product (GDP) values in combination with provincial forestry statistics to estimate the overall contribution of oak timber products to the eastern Canadian economy (Table 4). Based on this approach, the total value of oak-related timber products was CDN$24 million annually. Again, values were highest in the province of Ontario—which had a combination of high forest-related GDP and high oak abundance—followed by Québec, New Brunswick, Nova Scotia, and Prince Edward Island. If we consider this amount to be an annual benefit that would occur indefinitely in the absence of oak wilt (i.e., a perpetuity), we can calculate its present value by dividing by a reasonable discount rate (e.g., 4%). This results in a present value of CDN$600 million for ongoing oak-related contributions to the GDP.

**Table 3:** Merchantable oak timber volumes and values for provinces/regions in Canada.

Province/region	Gross Merch. Volume > 40 years, (m^3^ × 10^6^)	Stumpage fee (CDN$/m^3^)	Standing timber value (CDN$ × 10^6^)
Maritimes	0.54	21.49	11.6
Québec	1.36	17.84	24.3
Ontario^a^	7.77	11.60	90.1
Total			126.0

^a^Includes small amount of harvestable oak from neighbouring province of Manitoba.

**Table 4:** Estimates of provincial GDP related to oak timber products.

Province	Forest-related GDP^a^ (CDN$ × 10^6^)	Hardwood harvest^b^ (prop. of total harvest)	Oak abundance^c^ (as prop. of hardwood component)	Oak-related GDP (CDN$ × 10^6^)
Prince Edward Island	7.8	0.76	0.008	0.047
Nova Scotia	240.4	0.19	0.012	0.548
New Brunswick	594.8	0.36	0.008	1.713
Quebec	3518.2	0.18	0.007	4.433
Ontario	1855.7	0.25	0.039	18.093
Total				24.834

^a^Source: Natural Resources Canada^49^.

^b^Sources: Province of Newfoundland and Labrador^50^; Prince Edward Island Statistics Bureau^51^; Nova Scotia Deparment of Natural Resources^52^; Province of New Brunswick^53^; Ministère des Forêts, de la Faune et des Parcs^54^; and Ontario Ministry of Natural Resources and Forestry^55^.

^c^Calculated from national forest inventory grids (Beaudoin et al.)^13^.

## Discussion

Our findings indicate that suitable climate conditions currently exist in southern Canada (particularly in southern Ontario) for *B. fagacearum* and two of its principal dispersal vectors *C. truncatus* and *C. sayi*. The ranges of all three species are projected to expand to encompass much of the natural range of oak in eastern Canada within the next few decades, underlining the existential risk that oak wilt presents to many of the oak species in Canada. Our climate niche models identified little or no suitable climate habitat for *B. fagacearum* in the extreme southwestern portion of British Columbia, suggesting that *Q. garryana*—the only oak species in this region—may not be at risk of contracting the pathogen. We note, however, that it is currently not clear if the absence of *B. fagacearum* from the west coast of the United States (where significant oak resources exist) is due to limiting climatic factors or a lack of exposure, which would require long-distance movements of the pathogen from established eastern populations^5^. Our models also project low climate suitability for *B. fagacearum* in southern Manitoba. This could represent a refugium for *Q. macrocarpa*—the only oak species that currently occurs in this region. However, given significant uncertainties in climate change trajectories and biotic responses, such spatial details should be interpreted with caution.

Following common practice, our distribution models are based on known occurrence locations for our species of interest. While studies have shown that accurate climate envelope models can be generated from relatively few occurrence locations^56^, model accuracy ultimately depends on the extent to which the occurrence locations sample the full climatic range of the target species. This topic is particularly apropos in the case of *B. fagacearum*—a pathogen thought to be introduced to the United States in the late 1800s and whose geographic origins are still unknown^5^. Given this situation, we recognize that our occurrence data may underestimate the potential climatic range of this species in the United States and, consequently, that our distribution models may underestimate the potential range of *B. fagacearum* in Canada. Given that much of the natural range of oak in Canada was identified as suitable for *B. fagacearum* with the current (optimistic) input data, these potential data limitations may have relatively little impact on our overall findings. However, they do add uncertainty to fine-scale spatial predictions, such as the existence and exact location of oak refugia under future climate projections.

Our findings indicate that the introduction of oak wilt to eastern Canada would put hundreds of millions of dollars (undiscounted) in street trees and timber products at risk. Oaks represent a relatively minor component of street trees in eastern Canada, averaging about two trees per km of roadway across the 485 communities included in this study. Nonetheless, we estimated nearly half a million oak street trees across our study area, with a total cost of approximately CDN$350 million for removal and replanting. Not surprisingly, large urban centres such as Montréal (CDN$54 million) and Toronto (CDN$48 million) would be expected to experience the largest economic impacts in the event of an oak wilt outbreak. In the natural setting, existing oak volumes were valued at CDN$126 million based on current stumpage rates; while oak-related GDP—which aims to include all economic activity related to oak products—was estimated at CDN$24 million per year (or CDN$600 million when expressed as the present value of a perpetuity). We are not aware of other studies that have estimated potential economic impacts related to oak wilt in Canada. However, Haight et al.^57^ estimated $US18–60 million in costs related to urban oak tree removals resulting from oak wilt spread in the Minneapolis-Saint Paul metropolitan region. These figures are comparable to those reported here for street tree removal and replacement in large Canadian urban centres.

We note a number of caveats to our economic impact findings. First, our cost categories are clearly a subset of the full set of values associated with oak trees. Some of the costs that we did not account for include oak trees in rural areas (e.g., near roads and dwellings), as well as urban trees located away from roadways (e.g., backyard and park trees). Second, we did not consider the value of ecosystem services provided by oak trees—such as runoff control, shade provision, carbon sequestration, recreational benefits, wildlife habitat and food source, and many others (see Farber et al.^58^); these services are challenging to quantify, but represent real loses to material (and non-material) human interests when trees are lost. Furthermore, we did not incorporate a timeline of impacts based on oak wilt spread across our study area; such a timeline would allow future costs to be appropriately discounted per standard economic theory^59^. However, projecting *B. fagacearum* spread patterns would require significant assumptions regarding introduction events, natural spread patterns, and long-distance human-assisted movements—all of which are highly uncertain. In addition, we do not consider behavioral responses by actors in the marketplace to minimize and/or delay economic impacts; such efforts could include pre-emptive removal of susceptible wood volumes, post-attack salvage harvesting, and/or substitution of lost oak harvest volumes with other species^15^. Finally, our economic impact analysis does not include estimates for the management of oak wilt. These management costs are situation-dependent and highly variable and therefore difficult to predict^60^. Any efforts to stop transmission of oak wilt would add to our estimated costs.

Some of these caveats (i.e., incomplete accounting of potential costs) would lead to an underestimate of total economic impacts, while others (i.e., lack of discounting and adaptation behaviour) could lead to an overestimate. However, given the significant suite of values attributed to trees^61^, it is likely that our impact projections are underestimates overall. For example, a coarse indication of values related to CO_2_ sequestration, storm water runoff, and air pollution removal by oaks in eastern Canada can be obtained from i-Tree (https://www.itreetools.org/)—a suite of tools developed by the USDA Forest Service to estimate tree-related benefits. As input to this application, we roughly estimated the number of oak trees from gridded estimates of oak volumes and age classes across eastern Canada^13^, in combination with oak growth curves for the northern United States^62^ and oak-specific allometric equations^63,64^. This approach produced an estimate of 1.8 × 10^8^ oak trees in eastern Canada with an annual economic benefit of CDN$41 million—or CDN$1.0 billion when expressed as the present value of a perpetuity at a 4% discount rate. Though clearly coarse and somewhat subject to debate, we provide these numbers to give a rough sense of the significant oak values that exist outside the cost categories presented here.

## Concluding comments

Oak wilt has not yet been reported in Canada, though it has been detected within a kilometer of the Ontario-Michigan border. Our distribution models indicate that suitable climate conditions currently occur in southern Ontario for both *B. fagacearum* and *C. truncatus*, with much of the oak range in eastern Canada becoming climatically suitable within the next two decades.

Understanding potential economic impacts can assist managers in responding appropriately to an invasive threat. Here we identified potential oak wilt impacts for several cost categories, including approximately CDN$350 million in street tree-related costs and CDN$112 million in standing timber value. Alternatively, oak timber values can be expressed as a CDN$24 million contribution to annual GDP via the production and sale of finished timber products (or CDN$600 million when expressed as a present value of a perpetuity). These values, as well as others not considered here, such as carbon sequestration, runoff control, and pollution removal, indicate significant scope for oak wilt prevention and management efforts.

## Supplementary information

Supplementary Table S1.
